# Tumor-Derived Extracellular Vesicles: A Means of Co-opting Macrophage Polarization in the Tumor Microenvironment

**DOI:** 10.3389/fcell.2021.746432

**Published:** 2021-10-08

**Authors:** Theodore Reed, Jeffrey Schorey, Crislyn D’Souza-Schorey

**Affiliations:** Department of Biological Sciences, University of Notre Dame, Notre Dame, IN, United States

**Keywords:** extracellular vesicles, tumor microenvironment, intercellular communication, macrophages, polarization

## Abstract

Extracellular vesicles (EVs) are a heterogeneous population of membrane-bound parcels of bioactive proteins, nucleic acids, and lipids released from almost all cell types. The diversity of cargo packaged into EVs proffer the induction of an array of effects on recipient cells. EVs released from tumor cells have emerged as a vital means of communication and immune modulation within the tumor microenvironment (TME). Macrophages are an important contributor to the TME with seemingly paradoxical roles promoting either pro- or anti-tumoral immune function depending on their activated phenotypes. Here, we discuss the influence of tumor-derived extracellular vesicles on the functional plasticity of macrophages in tumor progression.

## Introduction

Extracellular vesicles (EVs) are a heterogeneous population of cell-derived vesicles secreted by virtually all cell types. They range in size from 15 nm to a few microns in diameter (D’Souza-Schorey and Schorey, 2018). EVs have been often been subclassified on the basis of size and/or their mode of biogenesis (Zijlstra and Di Vizio, 2018; Sheehan and D’Souza-Schorey, 2019). For example, exosomes are derived from the fusion of the multivesicular bodies with the plasma membrane and release of the intraluminal vesicles into the extracellular space, whereas microvesicles, which are larger than exosomes, are derived from outward budding and pinching of the plasma membrane. The international society for extracellular vesicles (ISEV) has provided the field with guidelines as well as nomenclature for the classification of EVs (Théry et al., 2018). The intracellular routes of EV biogenesis in many ways dictate the composition of EV subtypes. As such, various protein markers are associated with the different EV subclasses. For example, exosomes are typically identified by the presence of CD63, CD9, CD81, TSG101, and HSP70 whereas markers of microvesicles (MVs) are less well understood but can include integrin receptors, ARF6, VAMP3, and MHC Class I as well as other components of the endosomal recycling pathway involved in their formation (Clancy et al., 2019; Jeppesen et al., 2019; Sheehan and D’Souza-Schorey, 2019). Most EV subclasses are thought to be loaded with bioactive cargo ranging from nucleic acids, cytoplasmic proteins, metabolites, and components of lipid rafts (Sheehan and D’Souza-Schorey, 2019) and with the expanding knowledge of the intracellular pathways that regulate EV loading (Clancy et al., 2019; Jeppesen et al., 2019; Lee et al., 2019; Kalluri and LeBleu, 2020), the mechanisms involved in cargo delivery remain an important and active area of investigation.

Over the past decade, EVs have emerged as important mediators of horizontal intercellular communication in both prokaryotes and higher eukaryotes, inducing a plethora of physiological processes and also disease pathologies (Boomgarden et al., 2020; and references therein). In the context of cancer, where EVs have been best characterized, many of the pathways leading to EV production are usurped (Clancy and D’Souza-Schorey, 2018; Kalluri and LeBleu, 2020). In fact, tumor cells have been thought to secrete EVs to a higher level compared to the normal parent populations and further the amounts shed increase with disease stage (Ginestra et al., 1998; Bebelman et al., 2018; and references therein). The removal of EVs from circulation limits tumor growth and metastasis, substantiating the importance of EVs in tumor progression (Bobrie et al., 2012; Peinado et al., 2012; Ortiz et al., 2019; Wortzel et al., 2019). There is burgeoning interest in how these vesicles may affect recipient cells in the tumor microenvironment (TME) including cancer-associated fibroblasts, cells of the tumor vasculature and infiltrating immune cells. In particular, the effects of EV signaling on immune responses has garnered increasing attention. The functions of T and B lymphocytes, macrophages, natural killer (NK) cells, monocytes, dendritic cells, neutrophils, and myeloid-derived suppressor cells (MDSCs) are all affected by EV signaling, and depending on the status of the immune cell type, EVs might trigger adaptive immune responses or suppress inflammation (Théry et al., 2009; Sheehan and D’Souza-Schorey, 2019; Droste et al., 2020).

Macrophages are one of the most abundant immune cell types found in the TME (Gleave et al., 1991). These professional phagocytes are a part of the innate immune response and some of the first cells to arrive at the site of tissue damage or infection. Macrophages aid in clearing damaged cells and tissues while simultaneously recruiting and activating other immune cells through various mechanisms including release of cytokines and chemokines, and antigen presentation (Unanue et al., 1976). In addition to these well-known functions, macrophages are also known for secreting angiogenic factors, aid in tissue repair and remodeling, and secreting growth and migration factors (Lin et al., 2002; Wyckoff et al., 2004). While the ability of macrophages to play a role in both clearing and repair is vital to the innate immune response, in the context of tumor associated macrophages (TAMs) these roles can in fact facilitate tumor progression. Indeed, macrophages can enhance intravasation, basement membrane degradation for successful invasion, and even support tumor cell migration (Lin et al., 2002; Wyckoff et al., 2004). Macrophages within the TME have been shown to engage in paracrine activating loops wherein macrophages secrete epidermal growth factor (EGF) and matrix metalloproteinases (MMPs) in response to the secretion of colony stimulating factor 1 (CSF-1) from tumors (Lin et al., 2002; Wyckoff et al., 2004; Goswami et al., 2005; Cardoso et al., 2014). As described below, tumors have demonstrated the ability to co-opt macrophage functions in order to promote tumor progression and this modulation of macrophage function is mediated, at least in part, by EVs. In the following sections, we discuss the mechanisms by which tumor-derived EVs affect macrophage activation and its impact on tumor progression, and begin with an overview of tumor-associated macrophage (TAM) polarization.

## Macrophage Polarization in the Tumor Microenvironment

**FIGURE 1:**
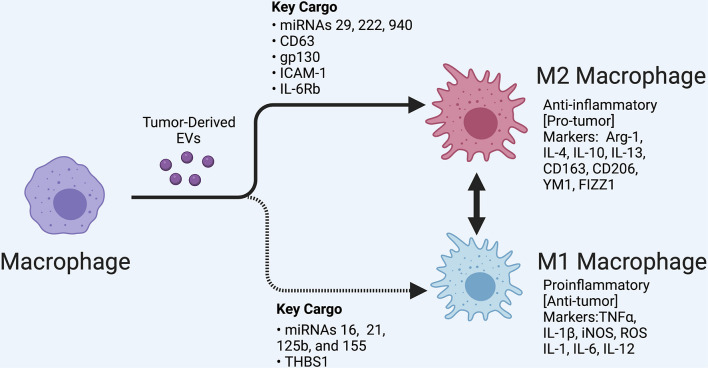
Macrophage polarization regulated by tumor-derived extracellular vesicles (EVs). A variety of tumors release EVs that can modulate tumor-associated macrophages depending in part on their cargo, as shown. M1 and M2 subsets are shown along with classifying cell markers. EVs can also regulate the transition between these polarization states. In the tumor microenvironment, polarized states across a spectrum are likely observed. Created with BioRender.com.

Tumor-associated macrophages have been shown to consistently react to signals received from tumors, including directly responding to tumor-derived EVs which can trigger multiple forms of macrophage activation and polarization (Arkhypov et al., 2020). Macrophages are activated by a myriad of factors that cause them to differentiate from their original monocyte progenitor. Once differentiated, macrophages undergo a secondary process called polarization, where they can take on either a proinflammatory phenotype traditionally referred to as M1, or an anti-inflammatory phenotype which is traditionally referred to as M2 (Figure 1). Macrophages are able to transition between these states depending on the signals they receive (Labonte et al., 2014). In the TME, this polarization and phenotype shift can be facilitated by EVs.

M1 macrophages produce factors associated with inflammation such as TNFα, IL-1β, iNOS, reactive oxygen species, IL-1, IL-6, and IL-12 (Labonte et al., 2014). Akt2 and PI3K-mediated signaling are major drivers of M1 polarization in various cancer models (Fang et al., 2004; Arranz et al., 2012; Labonte et al., 2014; Wang et al., 2014). Additionally, the transcription factor IRF5 has been shown to be active in proinflammatory macrophages (Vergadi et al., 2017). Another common feature of M1 macrophages is inflammasome activation. The inflammasome is involved in cleavage of pro-IL-1β resulting in its release from macrophages in its activated form (Lopez-Castejon and Brough, 2011). Although M1 macrophages are primarily considered anti-tumorigenic, their potential to induce a chronic inflammatory response can promote tumor progression as discussed later in this review.

Alternatively activated macrophages, also referred to as M2 macrophages, are associated with an anti-inflammatory and immunosuppressive response (Aminin and Wang, 2021). The M2 phenotype typically arises when the cause of tissue damage has been cleared and the immune response is no longer required. The M2 phenotype can be induced directly from a circulating monocyte or by conversion of an M1 macrophage (Evans et al., 2018). The presence of M2 macrophages in the TME provides a means to limit or divert the immune response (Shields et al., 2020). Activation of Arg-1, IL-4, IL-10, IL-13, CD163, CD206, YM1, and FIZZ1 are typically used as indicators of M2 polarization (Labonte et al., 2014). There are several known signaling molecules associated with this alternate macrophage activation including PI3K/Akt1 (Arranz et al., 2012; Vergadi et al., 2017). Further, IRF4 activation has been shown to contribute to the immunosuppressive macrophage phenotype (Wang et al., 2014).

## M1 Polarization-Mediated by Extracellular Vesicles

Since EVs contain a multitude of bioactive cargos, it is not surprising that their release from tumors have the capacity to modulate how macrophages respond in the TME. In several different types of cancer including oral squamous cell carcinoma (OSCC), brain cancer, pancreatic ductal adenocarcinoma (PDAC), and colorectal cancer (CRC), tumor cells have been shown to directly trigger M1 polarization through their secreted EVs (Jang et al., 2013; Su et al., 2016; Shao et al., 2018; Xiao et al., 2018). Investigations have begun to elucidate a list of bioactive cargos contained within EVs that can trigger this M1 polarization in recipient macrophages. The glycoprotein thrombosondin-1 (THBS1) and miRNAs 16, 21, 125b, and 155 have been shown to be transferred to macrophages through tumor EVs and induce M1 polarization in OSCC, breast cancer, PDAC, and CRC, respectively (Jang et al., 2013; Su et al., 2016; Shao et al., 2018; Xiao et al., 2018).

M1 macrophages release various factors that can induce tumor cell death, recruit and activate additional immune cell types, and directly remodel or degrade the surrounding tissue (Najafi et al., 2019). However, while classically activated macrophages are typically considered “anti-tumor,” some functions may in fact promote cancer progression. As example, immune cell recruitment can trigger inflammation in the TME and can stimulate vasodilation (Pober and Sessa, 2014) resulting in increase in blood vessel leakage and local angiogenesis creates an opportunity for tumor cells to gain access to the vascular space and thus contribute to metastatic potential. In the study performed by Xiao et al. (2018), exosomes loaded with THBS1 from OSCC were able to increase the transcription and subsequent protein secretion of typical the M1 markers TNF-α, IL-1β, and IL-6. Further, this same study showed that when conditioned media collected from macrophages previously treated with OSCC-derived EVs was added to OSCC cells it resulted in a statistically significant increase in OSCC cell migration relative to OSCC cells exposed to condition media from untreated macrophages (Xiao et al., 2018). Similarly, Shao et al. (2018) showed that small extracellular vesicles (sEVs) derived from CRC established an inflammatory, premetastatic niche for liver metastasis in part through the polarization of macrophages to an M1 phenotype. MiRNA-21 in sEVs was shown to stimulate M1 macrophages through a TLR7-mediated pathway (Shao et al., 2018). This inflammation, attributed to M1 activated macrophages, was confirmed in a retrospective study performed on patient serum samples, ultimately linking metastasis to the upregulation of proinflammatory cytokine, IL-6 (Shao et al., 2018). Another study performed in a breast cancer model found that 4T1 breast cancer cells when treated with epigallocatechin gallate (EGCG), a catechin with known anti-tumor effects, released EVs containing miRNA-16 (Jang et al., 2013). Exosomal miRNA-16 prevents TAM infiltration and inhibits M2 polarization. The study showed that miRNA-16 mediated this activity by down regulating IKKα resulting in the suppression of NF-κB and accumulation of Iκ-B. Similar findings of were made in several other studies pertaining to M1 polarization of TAMs and are summarized in Table 1.

**TABLE 1:** Tumor-derived extracellular vesicles (EVs) and their effect on macrophage polarization based on their cargo.

**Polarization**	**Tumor source of EVs**	**Key cargo**	**References**
Ml	Oral squamous cell carcinoma (OSCC)	THBS1	Xiao et al., 2018
	Breast cancer	MiRNA-16	Jang et al., 2013
	Pancreatic ductal adenocarcinoma (PDAC)	MiRNA-155, miRNA-125b	Su et al., 2016
	Colorectal cancer (CRC)	MiRNA-21	Popēna et al., 2018; Shao et al., 2018
M2	Triple negative breast cancer (TNBC)	CD63	Piao et al., 2017
	Hepatocellular carcinoma (HCC)	LncRNAs	Li et al., 2018
	Lung cancer	MiR-103a, TRIM59	Hsu et al., 2018; Liang et al., 2020; Pritchard et al., 2020
	Epithelial ovarian cancer	MiRNA-222	Ying et al., 2016
	Glioblastoma (GBM)	Focal adhesion proteins, leukocyte transendothelial migration proteins, miRNA-1246	Gabrusiewicz et al., 2018; Qian et al., 2020
	Oral squamous cell carcinoma (OSCC)	MiRNA-21, miRNA-29a-3p	Li et al., 2016; Cai et al., 2019
	Colorectal cancer (CRC)	MiRNA-203, miRNA-934	Takano et al., 2017; Wang et al., 2020; Zhao et al., 2020

## M2 Polarization-Mediated by Extracellular Vesicles

M2 polarization is deemed “pro-tumor,” as it has the ability to reduce inflammation and diminish immune cell activity through secretion of cytokines and other inhibitory factors (Aminin and Wang, 2021). Tumors are known to evade the immune response through several mechanisms, including recruiting/inducing tumor infiltrating lymphocytes (TILs) which have an immunosuppressive phenotype, such as M2 macrophages, T_*reg*_ cells, and T_*H*_17 cells (Sakaguchi et al., 2008). EVs released from tumors, when taken up by immune cells in the TME can be the direct cause of this immune suppression. This suppression of the immune system allows tumors to evade detection and subsequent destruction. It also allows for tumor expansion through cell proliferation, which also facilitates generation of additional protumor mutations over time. In addition, expression of various checkpoint inhibitors by tumor cells can suppress the anti-tumor immune response (Russell et al., 2021). Checkpoint inhibitors on EVs released from tumors can drive this immune suppression by serving as decoys (Lawler et al., 2020). Chen et al. (2018) identifies PD-L1 on EVs using *in vivo* models as well as patient serum and showed EV PD-L1 interacting with PD-1 on CD8 T cells in a manner that inhibited T cell cytotoxic function. PD-1 has also been shown to be expressed on TAMs and is associated with increased phagocytosis, reduction of tumor size, and increased survival in mice (Gordon et al., 2017). Previous studies have shown that specific EVs can upregulate PD-L1 expression on a variety of immune cell types, including neutrophils (Zhang et al., 2020) and myeloid cells (Fleming et al., 2019). This phenomenon is also observed in macrophages. In a more recent study, Liu et al. (2015) showed that HCC cancer cells released EV-associated miRNA-23a-3p in response to endoplasmic reticulum (ER) stress which led to an increase in macrophage PD-L1 expression (Liu et al., 2020). The study demonstrated an increase in PD-L1 on macrophage cell surfaces in patient samples as well as in *in vitro* studies. Interactions between immune checkpoint receptors and ligands are an important aspect of communication within the TME and can be modulated by the release of EVs or the presence of these ligands on EVs.

Extracellular vesicles-mediated M2 polarization of macrophages has been demonstrated in models of triple negative breast cancer (TNBC), hepatocellular carcinoma (HCC), lung cancer, prostate cancer, OSCC, epithelial ovarian cancer, glioblastoma (GBM), and CRC (Li et al., 2016, 2018; Chen et al., 2017; Piao et al., 2017; Takano et al., 2017; Gabrusiewicz et al., 2018; Hsu et al., 2018). Several EV-specific cargos have been linked to this alternate activation of macrophages, including miRNAs 29, 222, and 940, CD63, gp130, ICAM-1, IL-6Rb, proteins involved in focal adhesion, proteins involved in leukocyte transendothelial migration, and cytoskeleton-centric proteins (Baig et al., 2020). In a study conducted by Piao et al. (2017), TNBC cells release CD63-containing EVs which were able to induce macrophage polarization to an M2 phenotype both *in vitro* and *in vivo*. This polarization was shown to contribute to axillary lymph node metastases in an orthotopic breast cancer model by increasing the M2 to M1 ratio. Another study demonstrated that lung cancer-derived EVs can polarize monocytes toward an M2 phenotype, which subsequently increased the oncogenic effects of macrophages through the horizontal transfer of miRNA-103a. Recipient macrophages stimulated angiogenesis in this cancer model through the targeting of PTEN and activation of the PI3 kinase as well as STAT3 signaling pathways (Hsu et al., 2018). Another study by Linton et al. (2018) found that EVs derived from PDAC and containing ICAM-1 and arachidonic acid were able to trigger macrophages to polarize to an M2 phenotype and secrete pro-tumorigenic factors including VEGF, MCP-1, IL-6, IL-1β, MMP-9, and TNF-α. These factors are known to induce angiogenesis, lymphocyte recruitment and infiltration, tumor fibrosis, and metastasis in PDAC (Linton et al., 2018). Several additional studies, as summarized in Table 1, have also shown tumor-derived EVs to induce M2 polarization and tumor progression.

## Discussion

Macrophages are recipients of EVs released from tumors but their response is heterogeneous and dependent on the cargo of the tumor EV. EV-mediated intercellular communication within the tumor microenvironment has been shown to promote the production of tumor-promoting factors even in M1 macrophages that are classically categorized as “anti-cancer” (Pober and Sessa, 2014; Shao et al., 2018; Xiao et al., 2018). Macrophages are important in the TME based on their capacity to recruit immune cells, remodel tissues, and secrete angiogenic factors. Conditioning of macrophages mediated through EVs changes the TME in ways that can be advantageous for tumor growth and metastases. Understanding how tumor EVs co-opt macrophage function has the potential to uncover not only important information about EVs themselves but also helps broaden our understanding of tumor invasion and metastasis, potentially revealing new diagnostic or prognostic biomarkers or new targets for directed therapies. The field would also benefit from longitudinal studies pertaining to the effects of tumor derived-EVs on macrophages.

As early responders in the immune response, macrophages are extremely sensitive to their surrounding environments. M1 polarization is typically associated with clearing infections and promoting inflammation. As described above, when this function is controlled by tumors through EV secretion, macrophages characteristically referred to as anti-tumor can actually promote tumor invasion and dissemination through tissue remodeling and stimulating angiogenesis. M2 polarization has been a well-documented polarization state within tumors as this activation state aids in local immunosuppression. The conversion of M1 to M2 macrophages is well documented within the TME, and EV secretion from tumors, as described above, is a strong facilitator of this change. Thus, the M1 and M2 cell phenotype provide only a snapshot at any given point in time. Further, the phenotype of an activated macrophage is not necessarily an indicator of its function. Indeed, there is emerging evidence for even further subclassifications of macrophage polarization states as techniques such as single cell sequencing are applied to further probe the transcript and protein profiles of activated macrophages. As these profiles are revealed, new markers and subsequently new panels of assays will become available to assess macrophage content and function and better understand polarization heterogeneity. These new discoveries need to be applied to our understanding of EVs so as to paint a more complete picture of the role of EVs in modulating macrophage function within the TME.

While not discussed in this review, it is important to note that macrophages also secrete EVs within the TME that are able to drive or inhibit tumor progression (Goughnour et al., 2020; Zhang et al., 2020; Chang et al., 2021; Liu et al., 2021; Xavier et al., 2021). This cross-talk between tumors and macrophages, can result in complex paracrine and autocrine circuits that affect disease progression. Moreover, the majority of studies have done little to address the role of the different EV subpopulations. Macrophage polarization by EVs has been defined, almost exclusively through the study of either exosomes or largely undefined EVs. New insights into the specific roles of exomeres, microvesicles and oncosomes, in addition to exosomes, in macrophage polarization may generate a very different picture on the current TME/immune cell landscape. The effects of these different EVs and their cargo will require further investigation but the result of this work will provide a better understanding of how the immune system is regulated by and responds to the TME.

## Author Contributions

All authors listed have made a substantial, direct and intellectual contribution to the work, and approved it for publication.

## Conflict of Interest

The authors declare that the research was conducted in the absence of any commercial or financial relationships that could be construed as a potential conflict of interest.

## Publisher’s Note

All claims expressed in this article are solely those of the authors and do not necessarily represent those of their affiliated organizations, or those of the publisher, the editors and the reviewers. Any product that may be evaluated in this article, or claim that may be made by its manufacturer, is not guaranteed or endorsed by the publisher.

## Abbreviations

Arf6: ADP-ribosylation factor 6
Arg-1: arginase-1
CD: cluster of differentiation
CSF-1: colony stimulating factor 1
CRC: colorectal cancer
EGCG: epigallocatechin gallate
EGF: epidermal growth factor
EV: extracellular vesicle
GBM: glioblastoma
gp130: glycoprotein 130
HCC: hepatocellular carcinoma
HSP70: heat shock protein 70
ICAM-1: intercellular adhesion molecule 1
I- κ B: inhibitor of nuclear factor κ B
IL: interleukin
iNOS: inducible nitric oxide synthase
IRF: interferon regulatory factor
ISEV: international society of extracellular vesicles
MCP-1: monocyte chemoattractant protein 1
MDSCs: myeloid-derived suppressor cells
MMP: matrix metalloproteinase
miRNA: microRNA
MV: microvesicle
NF- κ B: nuclear factor kappa-light-chain-enhancer of activated B cells
NK cells: natural killer cells
OSCC: oral squamous cell carcinoma
PDAC: pancreatic ductal adenocarcinoma
PD-L1: programmed death ligand 1
PD-1: programmed cell death protein 1
PI3K: phosphoinositide 3 kinase
PTEN: phosphate and tensin homolog
RNA: ribonucleic acid
sEV: small extracellular vesicle
STAT3: signal transducer and activator of transcription 3
TAM: tumor associated macrophage
THBS1: thrombospondin-1
TIL: tumor infiltrating lymphocyte
TLR7: toll-like receptor 7
TME: tumor microenvironment
TNBC: triple negative breast cancer
TNF- α: tumor necrosis factor alpha
TSG101: tumor susceptibility gene 101
T_*H*_17: helper T cell 17
Treg: regulatory T cell
VEGF: vascular endothelial growth factor.
